# Foreign Substance in the Trachea

**Published:** 1881-02

**Authors:** 


AN interesting case of a foreign substance in the trachea is detailed in the *Nashville Journal of Medicine and Surgery*.

The patient, aged seventeen, while eating hickory nuts suddenly started to run and inhaled a piece into the trachea. Severe coughing ensued, but resulted only in failure to dislodge the substance, which was located after a time in the right bronchia, causing considerable cough and expectoration of mucus with occasion show of blood. The young man continued hale and hearty and well developed, and grew to full manhood doing ordinary labor, till suddenly profuse hæmorrhage ensued which was nearly fatal, but yielded to energetic treatment. From this time there was profuse discharge of pus, mixed more or less with blood. Eleven months subsequently while the young man was in bed, he had a severe spell of coughing, and felt something suddenly give way at the point of trouble, followed immediately by a sense of strangulation and impending death. After energetic efforts a large quantity of pus, largely mixed with blood, was discharged, and cause of all this suffering and danger found in the mass. A piece of hickory nutshell almost perfect with a piece of the kernel still in it, which dropped out when washed. The shell measured when dried 9-16ths of an inch on two sides and 7-16ths on the other, and weighed 10 grains, was convex on one side, concave on the other, and not apparently softened, though in the lung exactly three years and seven months. The young man continued to improve daily, and there is no reasonable doubt of his ultimate recovery.

THREE deaths from chloroform are reported as having occurred in November and December, one in Kentucky, one in London and one in Vienna.—(*New York Medical Record.*)

